# Supplementary material for: Are carbon emissions trading and green financial instruments synergistic? -Comprehensive quantitative research based on content analysis
Source: PLoS One. 2024 Mar 7;19(3):e0298601. doi: 10.1371/journal.pone.0298601 (PMC10919601; doi:10.1371/journal.pone.0298601)
Supplement: S1 Data — (ZIP) [file pone.0298601.s001.zip › Data/地级市Z_F工作报告.zip!/║■─╧/╓Ω╓▐╩╨/└┤╫╘╣1⁄2╓┌║┼í╛┬φ┐╦╩2╛▌═°í┐_2013.docx]

各位代表：

　　现在，我代表市人民政府向大会作工作报告，请予审查，并请市政协各位委员和其他列席同志提出意见。

　　过去五年的工作回顾

　　过去五年，是株洲经济社会发展极不寻常、极具挑战、极富成效的五年。五年来，我们在市委的正确领导下，紧扣“保二争一、科学跨越”战略目标，充分把握国家两型社会建设、中部崛起战略等重大机遇，有效应对国际金融危机，成功战胜雨雪冰冻灾害，克难奋进，开拓创新，完成或超额完成了十三届人民政府确定的各项目标任务，全市经济社会发展跨上历史性新台阶。

　　这五年，是加快转变发展方式、综合实力大跨越的五年

　　经济实力显著增强。2012年，全市预计实现GDP 1780亿元，增长13%,是2007年的2.4倍，年均增长14.1%;完成公共财政预算总收入213.8亿元，增长21.9%,是2007年的3.2倍，年均增长25.9%;完成固定资产投资1100亿元，增长35%,是2007年的3.8倍，年均增长37.3%;完成社会消费品零售总额580亿元，增长16%,是2007年的2.3倍，年均增长18.5%;城镇居民人均可支配收入和农民人均纯收入分别提高到25800元、11000元，增长15%、19%,是2007年的1.8倍、2.2倍。

　　产业转型加速推进。实施“5115”工程成效显著，南车株机、南车株所、株冶、唐人神4家企业年销售收入过100亿元；时代电气、株硬集团、株洲电业、南方公司4家企业过50亿元；天桥起重、唐人神、旗滨玻璃成功上市，上市企业达到10家。全力推进园区攻坚战役，轨道交通、航空、汽车等特色园区已具规模，各县（市）区的工业园区健康发展，北汽株洲、IGBT、高精传动、五矿工具等一大批战略性新兴产业项目和极具成长性的企业入驻园区，加速了“四城三基地”的构建和五大千亿产业集群的形成，全市规模工业增加值由2007年的282亿元增加到730亿元，年均增长19.3%.粮食总产量突破180万吨，连续九年丰收，农业机械化率达到64%,农产品加工产值突破200亿元，是2007年的2.6倍，唐人神集团成为全国首批农产品加工龙头企业。大力实施旅游升温战役，旅游年收入达到137亿元，是2007年的3.9倍，神农城、神农谷获评国家4A级旅游景区。南车物流、普洛斯物流等一批物流企业相继落户，希尔顿、喜来登、万豪、洲际等国际品牌酒店先后加盟，王府井等12家大型商场建成营业，获评福布斯中部最佳商业城市。新增中国驰名商标21件，被授予国家商标战略实施示范城市。

　　经济结构不断优化。2012年，高新技术产品增加值达到340亿元，是2007年的3倍，占全市规模工业增加值的比重达到46.6 %,万元规模工业增加值能耗累计下降57.3%,新型工业化考核连续五年获评全省一等奖；外贸进出口总额达到21.5亿美元，比上年增长15%,是2007年的2倍；引进外来资金359.5亿元，增长18%,是2007年的2倍；非公经济占GDP的比重达到56.7%.

　　这五年，是加快城市提质扩容、城市面貌大变样的五年

　　发展空间不断拓展。加快形成“一体三极”,依托市区主体，向北打造云龙新城，向西拓展武广、栗雨新城和新马组团，向南启动航空城、服饰城和枫溪生态城的建设，拓展了城市发展空间。加快构建“一圈三环”,芦淞大桥、田心立交、王家坪立交等相继竣工；株洲大道延伸段、炎帝大道、时代大道、云龙大道、迎宾大道全面建成，城市东西南北四张大门全部打开。衡炎、长株、炎睦、分炎等高速公路顺利完工，岳汝、垄茶等高速公路即将通车，市域2小时通勤圈基本形成。武广高铁投入运营，衡茶吉铁路、沪昆高铁、通用机场建设扎实推进，城市交通枢纽优势更加凸显。

　　城市品质全面提升。大力实施城市提质战役，推进“三创五改”、“四创四化”,城区面貌焕然一新；神农城、湘江风光带、华强文化科技产业基地、职教大学城“四大精品工程”进展顺利，神农城荣获中国风景园林学会金奖，湘江风光带河西段荣获国际风景园林师联合会大奖。第六次城市总体规划修改完成，规划展览馆、传媒大厦建成使用。推动城市管理重心下移，完善了“两级主体、分级管理”的城市管理体制，形成了“运营市场化、管理网格化、作业精细化、考核标准化”的城市管理模式，城市管理水平明显提升，株洲、醴陵、攸县分别获评全省最干净地级市、县级市和县城。成功创建国家园林城市、国家卫生城市、全国交通管理模范城市，创建国家环保模范城市26项指标全部达标，创建全国文明城市获得提名资格。

　　“智慧株洲”加快建设。促进工业化和信息化深度融合，轨道交通、有色冶炼、服饰等重点产业信息化改造步伐加快；“智慧株洲”顶层设计全面完成，科技创新体系、现代教育体系、信息应用体系日趋完善；依托“数字株洲”,搭建了应急指挥信息系统，建成了治安防控、智能交通、数字城管、数字环保系统；“三网融合”加速推进，基础网络日趋完善，被列为国家数字化城市管理试点城市。

　　这五年，是加快统筹城乡发展、新农村建设大提速的五年

　　县域发展明显加快。小城镇建成区面积从155平方公里拓展到215平方公里，常住人口增加到122万人，城市化率达到58 %.县城在县域经济中的首位度明显增强，县域经济占全市GDP的比重达到50.8%.醴陵市跨入全国百强县行列，攸县稳居全省十强县，株洲县、茶陵县、炎陵县被评为全省县域经济发展先进县。

　　基础设施不断完善。新建改建农村公路1.1万公里，实现了村村通水泥路。建成洮水水库，除险加固病险水库167座，完成湘江堤防工程17公里，整修加固支流堤防22.9公里，连续两年夺得全省水利建设“芙蓉杯”.新造林85.5万亩，森林覆盖率达到61.5%.完成农村改水改厕20万户，户用沼气发展到8.9万个，113个新农村示范村面貌明显改观。

　　发展活力得到增强。全面落实强农惠农政策，激活农村生产要素，发展农民专业合作社754家，农村土地流转率达到35%.启动523个撤村建居试点，基层组织管理体系进一步完善。云田、松西子、荷塘月色等成为全省新农村示范典型，高塘村城乡统筹模式在全省推广。攸县城乡环境同治经验走向全国。

　　这五年，是加快推进改革创新、两型建设大突破的五年

　　综合配套改革成效显著。坚持市场化导向，按照“资源资产化、资产资本化、资本证券化”理念，组建“7+2”投融资公司，在全省率先完成投融资平台公司化改造，为城市建设和产业发展提供了坚实支撑，确保了国有资产保值增值。推进城市建设体制改革，建立城市片区开发建设新模式，调动了城区和社会资本参与城市建设的积极性。大力推进城乡建设用地增减挂钩，探索推行“地票”交易，土地集约节约利用水平进一步提升。探索“政居分设、选聘结合”社区管理体制，完成了城区53个村改社区，基层管理体制更加顺畅。在全省率先实施排污权交易，环境保护和资源节约机制建设取得阶段性成果。

　　示范区建设全面铺开。云龙示范区着力发展高端服务业，云峰湖国际旅游度假区、总部经济园、北欧小镇、职教大学城等项目加快建设，“生态城、科教城、旅游城”初具规模。天易示范区把发展高新技术产业和建设现代化科技生态新城融为一体，积极构建两型产业体系，扎实推进风电产业园、新能源汽车、湘水湾生态园、栗雨中央商务区和新马组团的建设，株洲大道二期、栗雨休闲谷等一批重点工程相继竣工，“生态工业园、创新科技园、产业集聚区”初见成效。

　　两型环境加速构建。深入开展“蓝天碧水净土静音”行动，关停污染企业162家，城区拆除烟囱372根，市区空气质量持续保持国家二级标准。“一江四港”综合治理力度不断加大，完成251个湘江流域水污染综合整治项目，市区集中式饮用水源水质达标率100%,湘江株洲段水质持续保持Ⅲ类标准。完成生活垃圾焚烧发电项目主体工程，建成7座生活污水处理厂、4座生活垃圾处理场和1座工业废水处理厂，实现了污水处理县城全覆盖。城区公交车全部置换成环保电动车，建成全国首座电动公交城；投放自行车近2万辆，设置站点1020个，建成全国示范的公共自行车租赁系统，低碳交通、绿色出行成为市民的时尚选择。

　　这五年，是加快构建和谐社会、民生事业大改善的五年

　　社会保障不断加强。新增城镇就业26.6万人，新增农村劳动力转移就业28.6万人。城区最低工资标准提高至1160元；城镇居民养老保险、医疗保险全面实施，新型农村养老保险、合作医疗保险全面覆盖，在全省率先实施市区城乡低保统筹。加强物价监测和“菜篮子”工程建设，提质改造城区农贸市场54家，新扩建城镇标准蔬菜基地2万亩。高标准建成市儿童社会福利院和流浪未成年人救助保护中心，残疾人权益得到保障。住房保障体系全面建立，新增廉租房26070套、公租房15105套、经济适用房4235套，完成棚户区改造17646户，完成农村安居房建设和农村危房改造18657栋，解决了9.1万户中低收入家庭的住房困难；发放住房公积金贷款57.7亿元，惠及2.7万户居民家庭。成功创建国家创业型城市。

　　社会事业全面发展。深入推进“快乐德育、人文智育、阳光体育”,稳步实施《教育强市五年行动计划》和《城区基础教育三年攻坚计划》，城区中小学塑胶运动场改造全面完成，建成义务教育合格学校452所，新增学位近2万个，实现了适龄儿童“零失学”,上学难问题得到根本解决；被国家确定为“地方政府促进高等职业教育发展综合改革试点城市”,11所院校进入职教大学城发展。实施国家“863”、“973”等重点科技项目50项，申请专利突破2万件，获评国家知识产权工作示范城市；组建硬质合金、轨道交通2个国家重点实验室，中国工程院院士达到3名；第八次获评全国科技进步先进市。公立医院改革试点成效显著，基本药物制度全面实施，市中心医院、21个社区卫生服务中心、141个卫生健康服务室建成投入使用，社区卫生服务网络实现全覆盖。文化行政管理体制改革顺利完成，文化市场规范有序；社区文化活动中心、乡镇综合文化站、农家书屋建设全面完成。全民健身运动深入开展。市档案馆晋升为国家一级档案馆。

　　社会大局和谐稳定。深化“平安株洲”建设，连续两年公众安全感和满意度民意测评居全省第一，连续三次获得“长安杯”.加强和改进突发事件舆论引导和信访工作，信访总量逐年下降。“12345”市长热线和市长信箱处理群众咨询、建议和投诉16.9万件，办结率99.8%.各项工作扎实有效，获评全国食品药品监管先进单位、全国质量监督检验检疫先进单位、全国人口计生综合改革示范市、全国人民防空先进城市；荣获全国综治工作四连冠、全国双拥模范城市和全国民族团结进步模范集体五连冠。

　　与此同时，生产安全、交通安全、消防安全、民兵预备役等工作得到加强，妇女儿童、外事侨务、广播电视、气象、水文、市志、国防、红十字、老龄、对口支援等工作都有新进步。

　　五年来，我们切实加强政府自身建设，积极推进依法行政，认真执行人大及其常委会决议、决定，自觉接受人大、政协和社会各界的监督，五年共办结人大代表建议1436件，政协委员提案1375件，办结率、见面率、满意率均为100%.积极推进“法治株洲”建设，“五五”普法成效显著，荣获全国法制宣传教育先进城市。全面推行科学民主的决策机制、精简效能的执行机制、奖惩分明的激励机制、权责统一的问责机制，政府执行力和公信力进一步提升。大力开展行政审批制度改革，建成网上行政审批电子监察系统，开启行政审批“绿色通道”,减少审批事项69项，缩短办结时限1/3以上，行政效能不断提升。按照“一岗双责”党风廉政责任制的要求，十分注重制度建设，加强重点建设工程招投标监管，强化公共支出和专项资金的监察和审计，廉政建设取得明显成效。

　　2012年是本届政府的收官之年，我们按照市委“三个加快”的总体要求，重点抓了五项工作。

　　一是着力提升发展质量。新型工业化加速推进，完成工业总产值2150亿元，增长16.9%;园区实现工业增加值480亿元，增长16%;开工建设标准厂房115万平方米，建成60.7万平方米；中小企业服务热线开通，促进了中小微企业加快发展。自主创新能力持续增强，北汽株洲自主研发的发动机项目正式投产，实现产能10万辆；世界首台储能式电力牵引轻轨车辆、国内首台中低速磁悬浮列车成功下线，首架株洲产直升飞机“欧若拉”正式亮相。农业现代化稳步实施，全年完成农业总产值200亿元，增长4.3%;生猪规模养殖出栏比重达到65%,年出栏达到440万头。现代服务业加速发展，万豪、希尔顿酒店主体工程基本建成，华润万家建成营业，炎帝陵成为第八批国家级风景名胜区。

　　二是着力推进两型建设。云龙示范区方特梦幻王国开工建设，云龙大道、云峰大道建成通车。天易示范区栗雨工业园基本建成、新马工业园开发全面铺开。清水塘循环经济工业区霞湾港清淤、大湖治理工程全面完工，重金属污水综合处理厂投入运行，旗滨玻璃绿色搬迁正式启动。削减污染物排放总量完成国家下达的任务指标，万元GDP能耗下降5%,万元规模工业增加值能耗下降17%。

　　三是着力优化城乡环境。高速公路通车里程达到345公里，G106炎陵分路口至炎帝陵牌坊、洞株路株洲段等6条国省干线新建和改造全面完成，建成农村公路400公里，城乡交通网络日臻完善。城市提质深入推进，“四创四化”成果持续巩固，国家森林城市、国家健康城市创建工作全面启动。按照“四清三基两统一化”的要求，深入推进农村环境综合整治，城乡环境进一步改善。

　　四是着力打造智慧城市。实施部省级科技项目104项，新增市级以上企业创新平台9家，南车电机电力设备电气绝缘国家重点实验室正式揭牌，轨道工程机械测控系统省级工程技术中心通过综合评审。整合政府系统门户网站和子站群，协同及移动办公政务云平台正式启动，“数字食安”、“数字社区”、“数字地理空间框架”、应急指挥系统等项目扎实推进。

　　五是着力改善民生民利。坚持财力向民生倾斜，民生支出占公共财政预算支出的比重近70%,全面完成省、市实事工程。发放小额担保贷款3.48亿元，积极支持就业和扶持自主创业。坚持教育优先发展，小学扩容、初中创优、高中提质三大任务初步完成，为7.3万幼儿提供公益普惠性学前教育服务，义务教育均衡发展评估全省第一。城镇医疗保险和新型农村合作医疗参合率均达到98.1%;市区城镇居民最低生活保障线标准提高到370元。茶陵县、炎陵县成为全省率先启动公立医院综合改革试点县。“荷花家园”二期廉租房以及水竹湖等9个公租房项目全面启动，发放廉租住房补贴1358万元，有效满足了广大住房困难家庭需求。

　　五载砥砺奋进，五度春华秋实。过去五年，我们经历了风雨，也见证了彩虹；我们挥洒了汗水，也创造了荣光！五年丰硕成果的取得，是市委坚强领导的结果，是市人大和市政协监督支持的结果，是各民主党派、工商联、无党派人士、各人民团体参政议政的结果，是全市人民勤奋努力的结果。在此，我代表市人民政府，向各位人大代表和政协委员，向驻株部队官兵和全市人民，向关心、支持、参与株洲建设的各界人士，致以诚挚的谢意和崇高的敬意！

　　五年的成绩来之不易，积累的经验弥足珍贵。

　　一是始终坚持更新观念不动摇。我们紧扣“保二争一、科学跨越”战略目标，认真落实“五个敢于”思想，树立“抓城市就是从战略上抓发展”的思路，立足国际视野、战略思维、两型理念，先行先试，敢闯敢试，边干边试，发展路径更加清晰。

　　二是始终坚持创新发展不动摇。我们牢牢把握“以现代工业文明为特征的生态宜居城市”的发展定位，充分利用两型试验区的历史机遇，以市场化为导向，努力创新体制机制，全面铺开“十大改革”,深入实践城市经营“三化”理念，发展动力明显增强。

　　三是始终坚持务本求实不动摇。我们按照“办实事，抓落实”的要求，一年一个主题，一年一个目标，聚精会神谋发展，心无旁骛抓落实。认准的事就一以贯之，部署的工作就一抓到底，埋头苦干，真抓实干，确保发展目标不变、速度不减、质量不降，发展成效更加显著。

　　四是始终坚持民生优先不动摇。我们秉承“老百姓的幸福感就是我们的成就感”的理念，切实把人民群众最关心、最现实、最直接的利益问题放在突出位置，一切发展为了人民、一切发展依靠人民，在决策中更多地尊重民意，在投入上更多地倾注民生，努力让人民群众安居乐业。

　　五是始终坚持勤政廉政、依法行政不动摇。我们坚持把勤奋履职、廉洁从政作为干事创业的基本要求，注重在奉献中体现价值，在奋斗中实现梦想，努力做到激情工作、规范管理，勤政廉政、依法行政，切实维护政府良好形象。

　　同时，我们也深刻认识到，株洲经济社会发展中也存在一些困难和问题，主要是：经济总量不够大，均量不够高，质量不够优，主导产业对经济增长的支撑作用不强；新型城镇化和新型工业化不够协调，城市环境优势尚未充分转化为招商优势、产业优势；产业结构不尽合理，第三产业特别是生产性服务业等发展还不充分；社会管理和公共服务体系还不够完善，群众的一些利益诉求还不能得到很好的满足；等等。对此，我们将高度重视，认真研究，采取有力措施，切实加以解决。

　　未来五年形势和任务

　　今后五年，是株洲实现科学跨越的关键期，是两型社会建设的攻坚期，是全面建成小康社会的冲刺期。我们迎来了国家中部崛起战略、罗霄山脉连片扶贫开发和经济结构战略性调整等重大机遇。同时，我们也面临经济下行压力加大、创新能力不强、资源环境矛盾突出等多重挑战。我们将以更大的魄力、更足的干劲、更硬的举措，奋力开创株洲科学发展新局面。

　　今后五年政府工作的指导思想是：全面贯彻党的十八大精神，紧紧围绕主题主线，扎实推进“四化两型”建设，紧紧扣住“以现代工业文明为特征的生态宜居城市”的发展定位，突出“三个提升”,深入实施科教先导、产业转型、城镇带动、民生优先战略，努力建设智慧株洲、实力株洲、绿色株洲、幸福株洲。

　　今后五年全市经济社会发展的主要奋斗目标是：在全面完成“十二五”发展目标的基础上，实现“两个突破、两个翻番、两个建成”,即到2017年，全市经济总量突破3000亿元，人均GDP突破10000美元；公共财政预算总收入翻一番，城乡居民人均收入翻一番；全面建成小康社会，努力建成全国文明城市。

　　围绕上述指导思想和奋斗目标，突出抓好五个方面的工作：

　　（一）突出民生优先，全面建成小康社会。坚持以人为本，最大限度地增进民生福祉，让人民群众共享发展成果，重点实施“四大工程”.

　　一是实施收入倍增工程。实施积极的就业政策，鼓励多渠道、多形式就业，以就业促增收；加强创业服务工作，支持高校毕业生到中小企业、非公有制企业、城乡基层就业和自主创业。落实支农惠农政策，拓宽农民增收渠道，促进农民长效增收；完善收入分配统筹机制，积极引导企业建立工资增长机制，不断提高企业职工劳动报酬。

　　二是实施社会保障工程。全面整合城乡居民基本养老保险和基本医疗保险，提高城乡居民社会保障水平；完善社会救助体系，增强社会救助能力，不断提高最低生活保障水平；提高住房公积金制度覆盖率，加强保障性住房建设和管理，加快发展公共租赁住房，抓好棚户区、城中村和农村危房改造，着力解决中低收入家庭住房困难。

　　三是实施健康惠民工程。加快发展医疗卫生事业，推进基本医疗保障普及化、医疗卫生机构建设规范化、城乡公共卫生服务均等化；构建市级医疗中心、区域医疗中心、社区卫生服务机构的市区医疗服务体系，以及县级公立医院、乡镇卫生院、村卫生室的农村三级医疗卫生服务体系，有效缓解人民群众看病难、看病贵问题，加快创建国家健康城市；建立健全食品药品供应和安全保障体系，努力创建食品药品安全示范城市。

　　四是实施文化提升工程。大力发展文化事业，壮大文化产业，加强市场监管，规范文化市场，繁荣艺术创作，激发文化活力，打响炎帝文化、红色文化和工业文化品牌。构建完善的公共文化服务体系，突出抓好图书馆、博物馆、科技馆、群众艺术馆、神农大剧院、神农艺术中心、青少年活动中心、妇女儿童发展中心、夕阳红文化艺术中心等一批文化生活服务设施建设，努力让广大市民享受更高层次、更具品位、更加丰富的文化娱乐生活。

　　（二）突出产业转型，构建现代产业体系。以提高经济质量和效益为中心，大力发展先进制造业和生产性服务业，逐步退出重化工业，着力构建产业结构优、创新能力强、开放程度高的现代产业体系，提升产业核心竞争力。

　　一是壮大产业规模。大力推进“5115”工程，落实“三特”和“三优”政策，支持南车株机、南车株所、北汽株洲、南方动力、唐人神、五矿株硬、时代新材、千金药业等企业做大做强；推进产业集群发展，加快建设“四城三基地”,全力打造轨道交通、航空、汽车、服饰、陶瓷五大千亿产业集群；强化产业集群配套效应，继续在延伸产业链、扩大本地配套等方面狠下功夫；十分关注非公经济发展，引导中小民营企业集群聚力、抱团取暖，促使主机企业与配套企业共同发展。继续实施园区攻坚战役，推进特色产业园区建设，大力发展总部经济，打造园区核心竞争力，力争把醴陵经济开发区建成国家级经济技术开发区。

　　二是优化产业结构。改造提升有色冶炼、化工、陶瓷、烟花等传统优势产业，逐步退出重化工业，大力发展以先进装备制造业为主体的高新技术产业，培育壮大新能源、新材料、电子信息、生物医药、文化创意、环保等战略性新兴产业，加快构建现代工业体系。大力发展生态、休闲、体验、观光等现代农业，提升粮食、生猪、花卉、果蔬等优势产业，壮大唐人神、好棒美、云田花木、神农粮油等骨干企业，加快构建新型农业产业体系。加快建成南车物流、芦淞服饰物流仓储配送中心、普洛斯物流、铜塘湾港区等物流基地，打造面向全省、辐射中南地区的区域性物流中心；加快神农谷国际文化旅游度假区和“一城三湖”景区开发建设，打造全国知名旅游目的地；推进金融服务创新，积极发展信息咨询、电子商务、服务外包等新型服务业，加快构建现代服务业发展体系。

　　三是提升产业质量。进一步优化产业投资结构，引导资本投向高新技术产业和战略性新兴产业，促进产业绿色发展；严格产业准入，提高招商引资质量和效益，重点瞄准国内外500强、行业50强和央企，引进战略投资者；积极开拓国内外市场，加大轨道交通装备、刀片刀具、陶瓷等品牌产品的出口力度，实现“引进来”和“走出去”同步发展；促进产业创新发展，努力提高产品科技含量和附加值，力争高新技术产业增加值占全市规模工业比重达到60%以上；深入实施节能减排全覆盖工程，坚决淘汰落后产能，促进产业低碳发展。

　　（三）突出城镇带动，推动城乡统筹发展。树立“全域株洲”理念，按照“集约、智能、绿色、低碳”的新型城镇化要求，加快城镇提质扩容步伐，增强城镇集聚和辐射能力，努力形成城镇相连、交通便捷、就业充分、生态宜居的城乡一体化发展格局。

　　一是科学规划城镇布局。强化规划龙头作用，按照“一主两次、两轴四带”的发展构想，以市区为主中心，以醴陵、攸县为次中心，沿岳汝高速、106国道完善市域城镇体系。确立城区沿江发展战略，做好湘江两岸城市景观设计，引导湘江两岸同步向南拓展。坚持“内提外拓、统筹发展”,大力推进云龙新城、武广新城、枫溪新城等“十大新城”建设，努力构筑“一体三极”、“一圈三环”的城区发展新格局。强化乡村规划编制和建设管理，努力解决农村有新房无新村的问题，把云田、高塘、松西子、荷塘月色等一批乡村建设成为新农村的典范。

　　二是完善城镇交通体系。加快公路交通网络建设，建成长株潭南环线和醴茶、炎汝、垄茶等三条高速公路，形成“五纵六横”高速公路架构和“十纵十八横”国省干线公路体系。改造提升醴茶铁路，建成衡茶吉铁路、长株潭城际铁路株洲段，进一步完善铁路路网。推进株洲通用机场、炎陵通用机场建设，抢占中南地区通用航空先发优势。加快城市客运枢纽站场建设，完善城镇客运基础设施。

　　三是提升城镇品质品位。抓好城镇污水处理、停车场所、公交站场、消防设施、垃圾处理、排渍站等市政设施建设，健全电力、自来水、燃气、成品油等供应保障体系，完善城镇功能。强化建筑精品意识，改善城镇景观形象，推广城区“四创四化”经验，巩固城乡环境同治成果，健全城镇管理长效机制，提高城镇现代化水平。

　　四是加快县城建设步伐。县城建设要增强城市意识，积极探索城关镇镇改市的路径，让县城积聚更多的生产要素，有更完备的基础设施，通过园区建设，拓展产业发展空间，吸纳更多的农村剩余劳动力有序转化为市民，走出一条有株洲特色的新型城镇化路子。

　　（四）突出两型引领，推进生态文明建设。树立尊重自然、顺应自然、保护自然的发展理念，着力推进绿色发展、循环发展、低碳发展，创建国家森林城市，建设美丽株洲。

　　一是全面促进资源节约。完善节能降耗考核体系，降低能源消耗强度，提高能源利用效率。巩固“低碳交通、绿色出行”建设成果，完善电动公交和公共自行车租赁系统，构建节能健康的公共交通体系。严守耕地保护红线，加强水资源循环利用，提高水、土地等资源的集约节约利用水平。

　　二是加大生态环境保护力度。突出抓好“一江四港”综合治理，加大工业和生活污水处理力度，基本消除烟囱，基本关停污染企业，基本解决污水直排湘江问题。加强饮用水源沿线生态保护，全面完成湘江株洲段河道采砂整治，抓好生态绿心开发保护，让人民群众喝上干净的水、呼吸上清洁的空气。

　　三是推进示范区建设。用好、用足、用活两型社会建设试验区的政策，结合实际，编制两型社会建设的指标体系，并一以贯之，形成株洲特色。加快云龙示范区高端服务业发展，打造全省乃至全国两型建设精品；加强天易示范区高新技术产业集聚，推进产城一体化建设；加速清水塘污染整治和重化工企业绿色搬迁，实现转型引领发展；努力把示范区打造成为高端服务业的先导区、高新技术产业的集聚区、转变发展方式的引领区。

　　（五）突出科教先导，加快建设创新型城市。增强科技创新能力，强化科技创新的引领作用，加快推进经济发展由要素驱动向创新驱动转变，努力建设创新型城市。

　　一是实施科技创新工程。强化企业创新主体地位，依托南车株机、南车株所、株硬集团、608所、千金药业等企业，建设一批国家重点实验室。加快科研成果转化，发展高新技术企业200家以上，争取实施50项以上国家“863”、“973”及国家重点专项计划，加大知识产权保护力度，实施品牌发展战略，力争科技成果转化率提高到90%以上，科技进步对经济增长的贡献率提高到60%以上。

　　二是实施教育提升工程。全面实施“快乐德育、人文智育、阳光体育”,推进义务教育均衡发展，职业教育优先发展，高等教育重点发展，幼儿、成人、特殊教育统筹发展，在全省率先建成教育强市。加快职教大学城建设步伐，积极构建职业教育“立交桥”,致力打造“中部地区职业教育创新之都”,建成株洲智慧谷。

　　三是实施信息应用工程。切实加快工业化与信息化融合步伐，建立健全制造、物流、交通、商业等产业智能体系，提升各类产业智能化应用水平。加速“三网融合”进程，促进物联网、电子商务广泛应用，构建高速、安全、健康的信息网络平台。提升“数字株洲”建设水平，加快数据中心资源整合力度，推进“数字社区”建设，强化市级基础平台统筹规划和集约建设，建立宏观经济与社会发展基础数据库。

　　2013年工作目标和任务

　　2013年，是贯彻落实党的十八大精神的第一年，是实施“十二五”规划的关键之年。抓好新一届政府的开局工作，责任重大，意义深远。今年全市经济社会发展的主要预期目标是：GDP增长12%以上；公共财政预算总收入和地方公共财政预算收入增长15%;全社会固定资产投资增长25 %;社会消费品零售总额增长16%;规模以上工业增加值增长16%;城乡居民收入增长15%;万元GDP能耗下降3.5 %以上，主要污染物排放总量完成省里下达的年度减排任务；城镇登记失业率控制在4.5 %以内；居民消费价格指数低于全省平均水平；人口自然增长率控制在7.8‰以内。

　　为实现以上目标，我们将按照“提升综合实力，提升生态水平，提升幸福指数”的总体要求，突出抓好十大产业项目、十大基础工程、十大民生实事，努力推动经济社会又好又快发展，为十四届人民政府开好头，起好步。

　　十大产业项目：1.南车株所IGBT建成投产；2.北汽株洲二工厂全面开工；3.中航高精传动基本建成；4.山河智能通用飞机主体完工；5.中国五矿精密工具产业园全面开工；6.华强方特梦幻王国建成开园；7.神农谷国际文化旅游度假区开工建设；8.华银攸县电厂并网发电；9.陶瓷文化创意产业园基本建成；10.唐人神1000万头生猪一体化工程全面实施。

　　十大基础工程：1.基本建成神农大剧院和神农艺术中心；2.全面开工建设湘江河东风光带；3.启动湘江七桥建设，全面开工湘江六桥；4.启动湘江大道和株潭南环线建设，醴茶、炎汝、垄茶三条高速建成通车；5.启动图书馆、科技馆建设；6.铜塘湾港区基本建成，投入运营；7.完成城际铁路主体工程；8.希尔顿、万豪酒店建成试营业；9.全面启动“一江四港”综合整治工程；10.启动天然气县域全覆盖工程。

　　十大民生实事：1.积极扩大就业。新增城镇就业5万人，实现“零就业家庭”动态援助100%,新增农村劳动力转移就业3万人。2.缓解就医难题。新型农村合作医疗参合率稳定在95%以上，城镇居民医疗保险住院补偿率稳定在70%以上。3.改善就学条件。新建、改扩建城区义务教育学校5所，新增义务教育学位7500个，资助家庭贫困学生2.4万人。4.加强住房保障。开工建设廉租住房1800套、公共租赁住房9000套、城市棚户区改造7000户。新增住房公积金建制人数1万人。5.完善社保体系。稳定新型农村养老保险参保率90%;新增企业养老保险参保人数3万人。6.保护生态环境。建成垃圾焚烧发电厂和餐厨垃圾处理场，城市污水处理率和垃圾无害化处理率分别达到90%、100%.7.确保饮水安全。启动城市安全饮水全覆盖工程，新解决14万农村人口饮水不安全问题。8.完善城市功能。加快建设青少年活动中心、妇女儿童发展中心、夕阳红文化艺术中心，建成10个社区服务中心。9.加强社会救助。市区城镇居民生活最低保障线标准提高到每月400元；新建和扩建农村敬老院6所。10.保障食品药品安全。建立全市统一的“数字食安”云平台，确保各类食品药品抽检合格率均达到95%。

　　全年重点抓好七个方面的工作：

　　（一）加快两型社会建设步伐。加强生态环境建设。深入推进“一江四港”综合治理，抓好新马、响塘、曲尺、枫溪等片区重金属污染土壤的生态修复和综合利用；启动枫溪污水处理厂建设，建成白石港、龙泉污水处理厂，提升城市治污能力；启动城市垃圾分类收集试点工作，加快生活垃圾焚烧发电厂建设。加快示范区建设。云龙示范区着力抓好云峰湖国际旅游度假区、云龙中央商务区等重点片区建设，全力推进职教大学城、总部经济园、智慧谷、数码科技城等重点项目建设；天易示范区重点启动太高产业园区建设，推进杨柳湖、汽配园、汽车主题公园等项目建设；清水塘循环经济工业区重点抓好重金属污染的生态修复和“三废”综合利用，积极推进旗滨玻璃、中盐株化等大型企业绿色搬迁，努力实现清水塘地区改造的历史性突破。全面推进两型企业、机关、学校、社区等示范创建工作。

　　（二）加速新型工业化进程。全力推进北汽株洲二工厂、南方宇航高精传动、湘煤集团煤机装备制造、五矿有色金属精深加工株洲基地、醴陵陶瓷艺术文化城及建筑陶瓷生产基地、渌鸿风力发电等项目建设；全面建成山河智能通用整机、攸县煤电一体化等续建项目。力争完成南车株所IGBT项目建设、日望动车组交流传动及网络控制系统技术改造；引导建立轨道交通、汽车及零部件、硬质合金、工程齿轮、陶瓷、服饰等产业技术联盟，推动中小企业集群发展。完成100万平方米标准厂房建设，提升园区承载能力。重点围绕支柱产业，加大科技创新力度，加快创新服务平台建设，引导企业增强知识产权意识，加大研发经费投入，提升企业的核心竞争力。

　　（三）提升农业现代化水平。推进农业产业化开发。加快中南农产品交易中心建设进度，启动中南水产品市场搬迁；组建湖南神农粮油发展集团，加快农产品加工园区建设；壮大松本林化、龙华牧业等企业，扶持特色花卉、油茶、茶叶等产业形成规模，力争全市农产品加工值突破230亿元。扩大农业规模化经营。加速农业机械化进程，加快实施粮食增产高效工程和农产品加工业振兴计划，确保粮食生产稳定在380万亩以上，生猪规模养殖比重提高到68%以上，蔬菜扩面超过1万亩，着力培育炎陵白鹅、茶陵黄牛、攸县麻鸭等农业品牌。大力推广农业科技。农业科技进村入户率提高到75%以上，科技进步对农业增长的贡献率提高到60%以上。夯实农村基础设施。启动村村通公交试点工程，除险加固病险水库89座，新建户用沼气池6000个。激发农村发展活力。全面推进百城千乡流通再造工程，进一步扩大农村消费需求。

　　（四）构建现代服务业体系。大力发展第三产业，着力推进云峰湖国际旅游度假区、荷塘月色、云阳山、东阳湖、酒仙湖、神农谷国际文化旅游度假区等重大旅游项目的深度开发，打造旅游发展品牌。加快南车物流、芦淞服饰物流仓储配送中心等项目建设，打造区域性物流中心。万豪、希尔顿酒店建成试营业，加快喜来登等其他国际品牌酒店建设步伐。加强房地产市场监管，促进房地产业健康发展。推进金融体制改革和服务创新，组建小额贷款公司和村镇银行，引导民间资本向“三农”和中小企业流动。

　　（五）推进城乡一体化建设。加快建设神农城、湘江风光带河东段等精品工程，提升城市品位；实施公交建设三年行动计划，启动建设一批公交车综合站场，完善城市功能。启动湘江七桥、渌枫大道、湘江大道建设，加快建设北环线、湘江六桥、机场大道、智慧大道、田心大道、荷塘大道和神农大道，进一步完善城市路网骨架；启动长株潭南环线、左权大道建设，加快建设铜霞路至京珠高速、S313老关至铁河口段等9条国省干线，建成衡茶吉铁路株洲段和醴茶、炎汝、垄茶3条高速公路，全面完成“十二五”农村公路通畅工程，进一步完善市域路网体系。深入推进城乡环境同治，按照“四清三基两统一化”的总体要求，深化农村环境综合整治，抓好一批示范片建设，提升示范带动作用。

　　（六）促进民生事业发展。优先发展教育事业。破解学前教育“瓶颈”,建设一批公办幼儿园，力争全市70%以上幼儿园达到三级以上建设标准，新建、改扩建城区义务教育学校5所；切实解决弱势群体子女、留守儿童教育管理问题。积极发展医疗卫生事业。巩固新型农村合作医疗成果，深化医药卫生体制改革，深入推进公立医院改革试点工作。继续办好“读书月”活动，多形式、多渠道丰富人民群众的文化生活，提升人民群众的文化素养。办好市第十二届运动会，丰富市民文体活动。加大困难群体就业援助力度，加快建设创业就业指导中心。加快保障性安居工程建设，健全廉租住房、公共租赁住房等多层次住房保障机制，满足中低收入家庭的住房需要。加强价格管理调控，确保物价稳定。认真抓好第三次经济普查工作。

　　（七）维护社会大局稳定。完善突发事件应急管理机制，全面提升基层应急救援能力。落实安全生产责任制，促进安全监管规范化、制度化，严防重特大事故发生。抓好芦淞市场群、合泰、龙泉“三合一”场所火灾隐患整治，切实保障消防安全。推进人防疏散基地及中心城区人防工程建设，健全人防防护体系。加强国防动员工作，夯实国防工作基础。严格落实信访责任，及时化解社会矛盾。巩固“平安株洲”创建成果，严厉打击各种违法犯罪活动，加强社会治安综合治理，确保社会和谐稳定。

　　为科学发展提供坚强保障

　　各位代表，实现新一届政府的目标任务，我们必须以更有效的举措、更优质的服务、更务实的政风，全面提升保障发展的能力和水平。

　　坚持改革引领。牢固树立“改革是最大的红利”意识，用改革的眼光、思路、办法解决前进中的问题。一是加快重点领域改革。纵深推进投融资、城区建设、医药卫生、资源节约和环境保护、城乡统筹发展等体制改革；按照“一权两制一司”的架构，进一步理顺园区管理体制，激发园区发展活力；加快农村集体土地确权、发证和流转步伐，积极应对营业税改增值税政策调整和分配体制改革，确保改革取得实效。二是深化行政体制改革。严格按照“经济调节、市场监管、社会管理、公共服务” 的职能要求，加快政府行政职能转变，深入推进科技、教育、文化、医疗卫生和事业单位改革，毫不动摇鼓励、支持、引导非公有制经济发展，探索基础设施和公共产品市场化运作机制，努力完善行为规范、运转协调、公正透明、廉洁高效的行政管理体制。三是加强和创新社会管理。强化户籍制度及配套改革，实施城乡统一的户口登记管理制度试点，加强特殊人群的服务管理，加快城市化步伐；大力推进农村改革，完善统筹城乡的社会保障、社会救助、医疗教育等制度，加快城乡一体化进程；深化“政居分设、选聘结合”的新型社区管理体制改革，夯实基层组织，提升社会管理水平。

　　注重“四化”融合。坚持走中国特色新型工业化、信息化、城镇化、农业现代化道路，促进工业化、信息化、城镇化、农业现代化同步协调发展。一是推动信息化和工业化深度融合。运用信息化手段改造提升有色冶炼、化工、陶瓷、烟花等传统产业，让这些传统产业更环保、更清洁、更现代化；运用现代信息技术提升装备制造业水平，推动“四城三基地”主导产业的高端化、国际化，形成多点支撑的现代工业体系。二是推动工业化和城镇化良性互动。城市是产业的载体，产业是城市的支撑，没有产业支撑的城市只是一座空城。要按照“工业园区化、园区城镇化”的要求，努力做到工业园区和城市新区同步规划，产业项目和配套设施同步推进，产业聚集效应和人口承载能力同步增强。三是推动城镇化和农业现代化相互协调。农业现代化是城镇化的强大推动力，城镇化为农业现代化提供了实现途径。要着力打造一批工业重镇、旅游名镇、商贸强镇和边界中心镇，积聚生产要素，发展农产品加工贸易，引导农民离土不离乡，稳步提高新型城镇化水平。

　　优化发展环境。坚持抓环境就是抓机遇、抓资本、抓发展的观念，大力推进环境建设，提升环境的“洼地效应”.以创新行政审批方式为突破口，加快电子政务建设，全力推进各类许可项目全部实现网上审批，构建务实高效的政务环境。以“法治湖南”建设为契机，加强法制宣传，规范行政执法行为，构筑公平正义的法治环境。以弘扬开拓进取、勇往直前的火车头精神为抓手，尊重首创精神，激发群众创造力，营造干事创业的人文环境。以诚信建设为基础，着力推广服务承诺制、行业执法公示制、生产经营信誉制，完善社会信用体系，建设诚实守信的市场环境。

　　加强政府自身建设。认真落实人大及其常委会的决议、决定，自觉接受人大及其常委会的监督，大力支持人民政协参政议政，认真办理人大代表建议和政协委员提案。强化执政为民理念，充分利用“12345”市长热线平台，认真倾听群众呼声，切实解决群众实际困难。加大政务公开力度，提高政府工作的透明度和公信力。改进工作作风，深入调查研究，精简会议文件，厉行勤俭节约，加强督促检查，狠抓工作落实。强化党风廉政建设，健全惩治和预防腐败体系，努力做到干部清正、政府清廉。

　　各位代表，新的事业催人奋进，新的使命崇高神圣。让我们在省委、省政府和市委的坚强领导下，凝聚全市人民的智慧和力量，抢抓机遇，开拓创新，同心同德，奋发图强，为全面建成小康社会而努力奋斗！
